# Autoantibody-mediated arthritis in the absence of C3 and activating Fcγ receptors: C5 is activated by the coagulation cascade

**DOI:** 10.1186/ar4117

**Published:** 2012-12-13

**Authors:** Jennifer L Auger, Stefanie Haasken, Bryce A Binstadt

**Affiliations:** 1 Center for Immunology and Department of Pediatrics, University of Minnesota, 2101 6th Street SE, Minneapolis, MN 55414, USA; 2 Current address: Iowa Inflammation Program, University of Iowa, 2501 Crosspark Road, D168-MTF, Coralville, IA 52241, USA

## Abstract

**Introduction:**

The effector functions of immunoglobulin G (IgG) are mediated by interaction of its Fc region with Fc receptors (FcγRs) and/or the complement system. The three main pathways of complement activation converge at C3. However, C3-independent pathways can activate C5 and other downstream complement components during IgG-initiated inflammatory responses. These C3-independent pathways of C5 activation are triggered by activating FcγRs in some systems or can be activated by factors of the coagulation cascade such as thrombin. Here we studied the interplay of C3, C5, and activating FcγRs in a model of spontaneous autoantibody-driven arthritis.

**Methods:**

We utilized the K/BxN TCR transgenic mouse model of arthritis. We bred K/BxN mice bearing targeted or naturally-occurring mutations in one or more of the genes encoding complement components C3, C5, and FcRγ, the cytoplasmic signaling chain shared by the activating FcγRs. We measured arthritis development, the production of arthritogenic autoantibodies, T cell activation status and cytokine synthesis. In addition, we treated mice with anti-C5 monoclonal antibodies or with the thrombin inhibitor argatroban.

**Results:**

We have previously shown that genetic deficiency of C5 protects K/BxN mice from the development of arthritis. We found here that C3-deficient K/BxN mice developed arthritis equivalent in severity to C3-sufficient animals. Arthritis also developed normally in K/BxN mice lacking both C3 and FcRγ, but could be ameliorated in these animals by treatment with anti-C5 monoclonal antibody or by treatment with argatroban. Production of arthritogenic autoantibodies, T cell activation, and T cell cytokine production were not affected by the absence of C3, C5, and/or FcRγ.

**Conclusions:**

In K/BxN mice, C5-dependent autoantibody-driven arthritis can occur in the genetic absence of both complement C3 and activating FcγRs. Our findings suggest that in this setting, thrombin activates C5 to provoke arthritis.

## Introduction

The ability of immunoglobulin and immune complexes, including autoantibodies, to provoke inflammation stems from the interaction of the Fc portion of antibody molecules with one or both of two major effector pathways: Fc receptors and the complement system. The relative contributions of these two pathways vary among different disease states and experimental systems [1-3]. A more detailed understanding of the mechanisms by which autoantibodies engage Fc receptors and complement to provoke pathology in a specific target tissue can permit a more tailored therapeutic intervention.

Fcγ receptors (FcγRs) recognize immunoglobulin G (IgG) and transduce either activating or inhibitory intracellular signals. In the mouse, the activating FcγRs include FcγRI, FcγRIII, and FcγRIV. The activating FcγRs share a common cytoplasmic signaling chain called FcRγ (encoded by the *Fcer1g *gene) responsible for signal transduction. Mice also express the inhibitory receptor FcγRIIB, whose cytoplasmic tail contains an inhibitory signaling motif. The outcome of an interaction of an FcγR-expressing cell with an IgG-containing immune complex depends on the relative expression levels of the various activating and inhibitory FcγRs and also the IgG subtype (for which the various FcγRs have differing affinities) [4].

**Figure 1:**
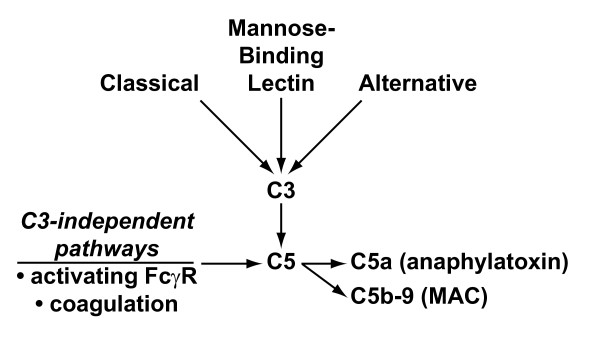
**Complement activation pathways**. The three traditional complement activation pathways converge at complement component C3, leading to the generation of a C5 convertase complex. Cleavage of C5 produces the anaphylatoxin C5a and C5b, initiating formation of the C5b-9 membrane attack complex (MAC). The present study focuses on C3-independent C5 activation pathways shown on the left: activating FcγRs and the coagulation cascade. FcγR, Fc receptor for immunoglobulin G.

The complement system is activated by three primary pathways (classic, alternative, and mannose-binding-lectin), each consisting of a series of serine proteases. These three activation pathways converge at complement component C3. Cleavage of complement C3 produces a C5 convertase. These events result in the generation of anaphylatoxins (for example, C3a and C5a) and formation of the membrane attack complex (C5b-9), whose main functions are to recruit inflammatory cells and to mediate cellular lysis, respectively (Figure 1) [5,6].

Several studies have pointed to the existence of additional, C3-independent mechanisms by which C5 can be activated to drive inflammatory responses (Figure 1). More than two decades ago, investigators described the existence of C5-C9-dependent immune hemolysis occurring in a C3-independent fashion [7,8]. More recently, studies of IgG-triggered acute lung injury revealed that, in C3-deficient mice, thrombin can act as a C5 convertase to generate C5a and mediate pathology [9]. Similar crosstalk between the complement system and coagulation systems has been identified in other model systems, including antiphospholipid antibody-induced and lipopolysaccharide (LPS)-induced fetal loss in mice [10,11]. An elegant *in vitro *study has recently confirmed that multiple serine proteases in the coagulation and fibrinolysis systems can cleave C3 and C5 to produce C3a and C5a [12]. Interplay of FcγRs and the complement system also occurs, and several studies of IgG-initiated pathology have highlighted the existence of a C5a generation pathway that is triggered by activating FcγRs [2,13-15]. Here, we investigated a possible contribution of C3-independent mechanisms of C5 activation in a mouse model of autoantibody-mediated arthritis.

K/BxN T-cell receptor (TCR) transgenic mice spontaneously develop inflammatory arthritis due to combined T- and B-cell recognition of the self-antigen glucose-6-phosphate isomerase (GPI) and production of high-titer anti-GPI IgG autoantibodies [16,17]. Arthritis can also be provoked by injecting serum from K/BxN mice into normal mice [18]. Importantly, the requirements for complement and activating FcγRs differ between the K/BxN TCR transgenic mice and its derivative, the serum transfer model, likely reflecting the several-fold higher concentration of anti-GPI autoantibodies in the spontaneous genetic model. The development of serum-transferred arthritis depends on both activating FcγRs and the alternative pathway of complement activation. Specifically, mice with targeted or naturally occurring mutations in the genes encoding factor B (of the alternative pathway), C3, C5, the C5a receptor (C5aR), and FcRγ were protected from developing serum-transferred arthritis [19-21]. In contrast, we have shown that K/BxN TCR transgenic mice lacking FcRγ developed spontaneous arthritis equivalently to controls but that C5-deficient K/BxN mice developed less severe arthritis than controls [22]. In addition, treating K/BxN mice with anti-C5 monoclonal antibody reduced their arthritis severity [22]. Those findings led us to investigate which of the upstream C5-activation pathways drives arthritis in K/BxN mice.

## Materials and methods

### Mice

KRN TCR transgenic mice on the C57BL/6 (B6) background [16] were a gift from Diane Mathis and Christophe Benoist (Harvard Medical School, Boston, MA, USA) and the Institut de Génétique et de Biologie Moléculaire et Cellulaire (Strasbourg, France). C5-deficient B6 mice congenic for the non-obese diabetic (NOD)-derived *Hc *allele (encoding non-functional C5) [22,23] and B6 mice congenic for H-2^g7 ^(B6.g7) were also a gift from Mathis and Benoist; I-A^g7 ^is the mouse major histocompatibility complex (MHC) class II molecule that presents GPI-derived peptides to activate KRN TCR-expressing T cells. *C3*-deficient mice on the B6 background [24] were a gift from Michael Carroll (Harvard Medical School). FcRγ (*Fcer1g*)-deficient mice on the B6 background [25] were purchased from Taconic (Hudson, NY, USA).

The C3-, C5-, FcRγ-, and double-deficient 'K/BxN' lines used in this study were created by breeding mice bearing the appropriate knockout allele(s) on the B6 background to KRN/B6 mice and also to B6.g7 congenic mice. The MHC (*H2*) is the only NOD-derived genetic region that the B6.g7 mice retain; to simplify nomenclature, however, we refer to the mice as 'K/BxN' throughout this study as we have previously [22]. Because *C3 *and the *H2 *complex both reside on mouse chromosome 17, a spontaneous chromosomal recombination event was necessary to generate *C3*-deficient B6.g7 congenic mice. Genotyping of mice was performed by standard polymerase chain reactions. Mice were bred in specific pathogen-free colonies under protocols approved by the University of Minnesota Institutional Animal Care and Use Committee.

### Antibodies

Anti-C5 monoclonal antibodies were derived from the BB5.1 hybridoma, a gift from Brigitta Stockinger (MRC National Institute for Medical Research, London, UK) [26]. Antibodies used for flow cytometry included anti-CD3 (clone 71A2), anti-CD44 (clone IM7), anti-CD62L (clone MEL-14), anti-interleukin-17 (anti-IL-17) (clone eBiol7B7), and anti-interferon-gamma (anti-IFNγ) (clone XMG1.2) from eBioscience (San Diego, CA, USA) and anti-CD4 (clone RM4-5) and anti-Vβ6 (clone RR4-7) from BD Pharmingen (San Diego, CA, USA).

### Assessment of arthritis, anti-C5 antibody treatment, anti-GPI titers, histology, and immunohistochemistry

Assessment of arthritis severity by clinical score and ankle thickening, treatment with anti-C5 antibody, determination of anti-GPI IgG titers by enzyme-linked immunosorbent assay, histological analysis, and immunohistochemistry for C3 and IgG were performed as described [22]. For detection of prothrombin, histologic sections of mouse liver were first blocked by using the avidin/biotin blocking kit (Invitrogen Corporation, Carlsbad, CA, USA). Primary antibodies then were added at a dilution of 1:20, in accordance with a prior report [9]. The primary antibodies were goat polyclonal IgG anti-thrombin (K20) or normal goat IgG (both from Santa Cruz Biotechnology, Inc., Santa Cruz, CA, USA). The primary antibodies were detected by secondary staining with biotin-coupled donkey anti-goat IgG diluted 1:2,000 (Santa Cruz Biotechnology, Inc.) followed by application of ImmPACT DAB peroxidase substrate with the ABC peroxidase kit (Vector Laboratories, Burlingame, CA, USA).

### Flow cytometry

Intracellular cytokine staining was performed in accordance with the instructions of the manufacturer (eBioscience). Flow cytometry was performed by using a FACSCalibur and an LSRII (BD Biosciences, San Jose, CA, USA), and cells were analyzed by using FlowJo V7.6 software (Tree Star, Inc., Ashland, OR, USA).

### Argatroban treatment

Argatroban (Sigma-Aldrich, St. Louis, MO, USA) was dissolved in dimethyl sulfoxide (DMSO) and injected intraperitoneally into mice at a dose of 9 mg/kg daily or five days per week fromthe ages of 3 to 6 weeks [27,28]. No differences were observed between those mice receiving argatroban daily and those receiving it five days per week, so the results were pooled for analysis. The vehicle control-treated animals received DMSO intraperitoneally.

## Results and Discussion

**Figure 2:**
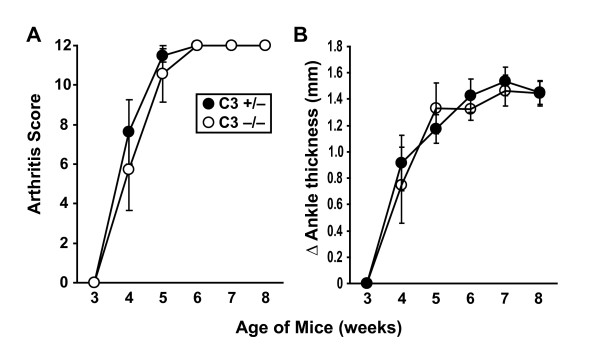
**Complement C3 deficiency has no effect on arthritis in K/BxN mice**. The development of arthritis in K/BxN T-cell receptor transgenic mice with a targeted deletion of *C3 *(open circles, *n *= 7 mice) and *C3*-sufficient littermate controls (filled circles, *n *= 8) was assessed by arthritis score **(a) **and ankle thickening **(b)**. Data are mean ± standard error of the mean. There are no statistically significant differences at any of the time points.

We recently reported that K/BxN TCR transgenic mice genetically lacking C5 or treated with anti-C5 monoclonal antibodies developed less severe arthritis than control animals [22]. To determine which of the upstream complement activation pathways were involved in arthritogenesis, we first investigated complement component C3, the convergence point for the three primary complement activation pathways and the component immediately upstream of C5. We bred K/BxN mice with a targeted deletion in the *C3 *gene. In agreement with a recent report, we observed that C3-deficient K/BxN mice developed arthritis equivalently to littermate controls (Figure 2) [29]. This finding suggested that a non-traditional, C3-independent pathway of C5 activation was at work in these mice.

**Figure 3:**
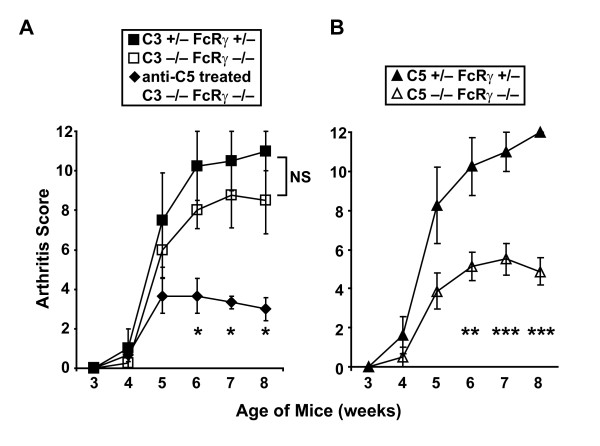
**C5-mediated arthritis in K/BxN mice occurs in the absence of C3 and FcRγ**. **(a) **Null alleles of *C3 *and *Fcer1g *(encoding FcRγ) were bred into the K/BxN mouse system. The development of arthritis was assessed in C3/FcRγ-deficient K/BxN mice bearing homozygous null alleles at both loci (open squares, *n *= 4) and in littermate controls expressing one wild-type allele at each locus (filled squares, *n *= 4). Data are mean ± standard error of the mean (SEM); there are no statistical differences at any of the time points for these two groups. Treatment of the C3/FcRγ-deficient K/BxN mice with anti-C5 antibody reduced arthritis severity (filled diamonds, *n *= 3); *P < 0.05 for the anti-C5 antibody-treated C3/FcRγ-deficient mice compared with untreated C3/FcRγ-deficient mice. **(b) **A naturally occurring null allele of *Hc *(encoding C5) and the targeted null allele of *Fcer1g *were bred into K/BxN mice. The development of arthritis was assessed in C5/FcRγ-deficient K/BxN mice (open triangles, *n *= 8) and in littermate controls with one wild-type allele at each locus (filled triangles, *n *= 8). Data are mean ± SEM; **P < 0.01; ***P < 0.001. FcRγ, the cytoplasmic signaling chain shared by activating Fc receptors for immunoglobulin G; NS, not significant.

Several reports have demonstrated that engagement of activating FcγRs can trigger C5a production, even in the absence of C3 [2,13-15]. We therefore hypothesized that, in C3-deficient K/BxN mice, the absence of activating FcγRs would impair C5 production and lead to less severe arthritis. To test this hypothesis, we bred K/BxN mice genetically lacking both C3 and FcRγ, the cytoplasmic signaling chain shared by the activating FcγRs. Surprisingly, the C3/FcRγ-double-deficient K/BxN mice developed arthritis of equivalent severity to controls (Figure 3a). As expected, immunofluorescent staining of joint sections from the C3/FcRγ-deficient K/BxN mice revealed IgG deposition similar to controls, but no C3 (Additional file 1a). Treatment of the C3/FcRγ-deficient K/BxN mice with anti-C5 monoclonal antibody reduced their arthritis severity (Figure 3a). Considered together, these findings indicate that, in the genetic absence of C3, arthritis in K/BxN mice can be mediated by an FcRγ-independent C5 activation pathway.

Consistent with our prior report that arthritis in K/BxN mice depends on C5 but not FcRγ [22], we found that K/BxN mice lacking both C5 and FcRγ developed less severe arthritis than controls (Figure 3b). In the ankles of these C5/FcRγ-deficient K/BxN mice, deposition of both IgG and C3 was detectable, suggesting that C3 deposition can still occur in the absence of C5 and despite the less severe inflammatory response in these joints (Additional file 1b). The fact that the C5/FcRγ-deficient mice developed some arthritis, albeit attenuated, suggests that other minor pathogenic effector mechanisms are still operational in these mice; candidate pathways include recognition of IgG immune complexes by upstream complement components and their receptors (for example, C1qR(P)/CD93 [30]), the pro-inflammatory activity of C3a [6], and pathogenic effector T cells.

**Figure 4:**
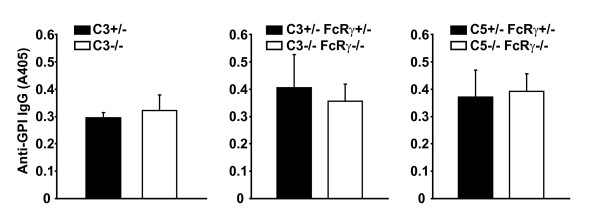
**Absence of C3, C5, and FcRγ does not impact autoantibody production in K/BxN mice**. Serum levels of anti-GPI IgG were determined in C3-deficient K/BxN mice and controls (left panel), C3/FcRγ-deficient K/BxN mice and controls (middle panel), and C5/FcRγ-deficient K/BxN mice and controls (right panel). Control animals are indicated by filled bars and knockout mice by open bars. A405 indicates absorbance in the enzyme-linked immunosorbent assay. Data are mean ± standard error of the mean. There are no statistically significant differences between the anti-GPI levels. FcRγ, the cytoplasmic signaling chain shared by activating Fc receptors for immunoglobulin G; GPI, glucose-6-phosphate isomerase; IgG, immunoglobulin G.

**Figure 5:**
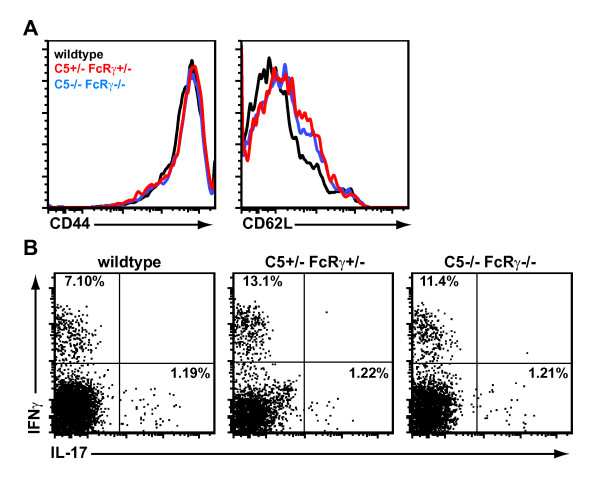
**T-cell activation and cytokine production are not affected by C5 and FcRγ deficiency in K/BxN mice**. **(a) **The plots depict cell surface expression of CD44 and CD62L on CD3^+ ^CD4^+ ^Vβ6^+^-gated splenocytes from the indicated mice. **(b) **Intracellular expression of the cytokines interleukin-17 (IL-17) and interferon-gamma (IFNγ) was detected in CD3^+ ^CD4^+ ^Vβ6^+ ^splenocytes from the indicated mice. Vβ6 is the TCRβ chain encoded by the KRN TCR transgene. Data are representative of two to six mice per group in three independent experiments. TCR, T-cell receptor.

We performed additional studies to determine how C5 deficiency interferes with arthritogenesis in K/BxN mice. We found that the production of anti-GPI autoantibodies was unimpaired in the C3-deficient, C3/FcRγ-deficient, and C5/FcRγ-deficient K/BxN mice (Figure 4). C5a was also recently shown to drive T helper 17 (Th17) cell differentiation in another murine arthritis model [31]. Th17 cells are known to be involved in the pathogenesis of arthritis in the K/BxN system [32,33]. We found, however, that T-cell activation as determined by high expression of CD44 and low expression of CD62L (Figure 5a) and the frequency of CD4^+ ^T cells producing the cytokines IFNγ or IL-17 (Figure 5b) were unaltered in the C5/FcRγ-deficient K/BxN mice relative to controls. Collectively, our findings indicate that the T cell- and B cell-dependent events culminating in autoantibody production and pro-inflammatory cytokine production are not impacted by deficiency of C3, C5, and/or FcRγ and suggest that C5 drives arthritis in K/BxN mice via downstream effector mechanisms. Given the requirement of the C5aR but not the terminal complement components (C6-C9) for serum-transferred arthritis [20], it is most logical that it is the interaction of C5a with C5aR that drives arthritis in K/BxN mice, a hypothesis that could be tested by generating C5aR-deficient K/BxN mice. Next, we investigated how C5 was being activated in the absence of C3 and activating FcγRs.

**Figure 6:**
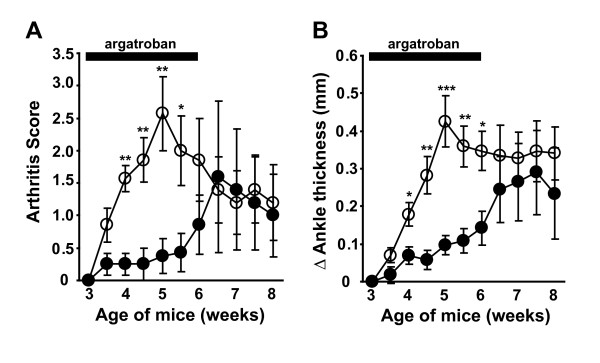
**Thrombin inhibition ameliorates arthritis in C3/FcRγ-deficient K/BxN mice**. K/BxN mice lacking C3 and FcRγ were treated with the thrombin inhibitor argatroban (filled circles) or vehicle control (open circles) from 3 to 6 weeks of age. Treatment was then stopped. The development of arthritis was determined by arthritis score **(a) **and twice weekly ankle measurements **(b)**. Data plotted are mean ± standard error of the mean and represent seven or eight mice per group compiled from three independent experiments. *P < 0.05, **P < 0.005, ***P < 0.001. FcRγ, the cytoplasmic signaling chain shared by activating Fc receptors for immunoglobulin G.

Multiple lines of evidence point to molecular communications between the coagulation and complement cascades, both of which contain numerous serine proteases. Administration of the anticoagulant heparin blocked antiphospholipid antibody-induced complement activation and pregnancy loss in a mouse model [10]. Similarly, LPS increased the expression of the membrane-bound prothrombinase FGL2, which can cleave C5 and which also has been linked mechanistically to LPS-induced abortions in mice [11,34]. In mice lacking C3, the expression of prothrombin was upregulated, and treatment of mice with anti-thrombin III blocked IgG-induced C5-dependent lung injury [9]. We verified that the expression level of prothrombin was increased in the liver of C3-deficient mice relative to control mice (Additional file 2). In keeping with this finding, treatment of C3-deficient/FcRγ-deficient K/BxN mice with the thrombin inhibitor argatroban led to reduced arthritis severity, and discontinuation of the argatroban was temporally associated with the re-emergence of arthritis (Figure 6). We are currently investigating longer-acting compounds to determine whether thrombin inhibition acts primarily to reduce arthritis severity or to delay the onset of arthritis in this model. Thus, the generation of C5 in the absence of C3 and activating FcγRs in K/BxN mice appears to be mediated by thrombin or related serine proteases of the coagulation cascade.

Whether C3-independent pathways of C5 activation contribute to pathology in wild-type mice or only in the setting of genetic C3 deficiency is important for understanding the relevance of these pathways to human diseases. For instance, heparin was effective in reducing antiphospholipid antibody-induced fetal loss in wild-type mice by blocking C5a generation [10]. Similarly, treatment with the thrombin inhibitor polyethyleneglycol-hirudin (PEG-hirudin) decreased the severity of collagen-induced arthritis in mice, although this was attributed to reductions in intra-articular fibrin deposition rather than to effects on complement activation [35]. In contrast, in the IgG-mediated lung injury model, the effect of the anticoagulants anti-thrombin III and hirduin in reducing lung pathology was evident only in C3-deficient animals, leading those investigators to speculate that genetic deficiency in C3 leads to upregulation of thrombin as a compensatory mechanism to allow C5 activation via a non-traditional pathway [9]. Similarly, we observed no effect of argatroban on arthritis severity in C3-sufficient K/BxN mice (data not shown), but we are currently exploring longer-acting thrombin inhibitors. Thus, the contribution of the coagulation cascade to C5 activation might vary depending on the disease model. In addition, a recent report suggests that, *in vitro*, thrombin cleaves C5 at a site different from that cleaved by C5 convertase, leading to the generation of novel intermediates [36]. Understanding whether these intermediates are also generated *in vivo *and, if so, how they affect inflammation will be essential next steps.

How IgG antibodies activate thrombin in the absence of activating FcγRs and C3 remains an open and important question. Since thrombin activation by IgG has been observed in multiple autoantibody-dependent models, it is not likely that the antigenic specificity is critical. It seems more likely that other IgG-interacting molecules (for example, complement C1q or the neonatal Fc receptor) could be at play. Alternatively, antibody fragments with direct prothrombinase catalytic activity have been described [37].

From a clinical perspective, monoclonal antibody reagents designed to interfere with C5 activation systemically or locally (for example, in the synovium) might be effective treatments for inflammatory arthritis (Figure 3a) [22,38,39]. Our findings suggest that agents designed to interfere with non-traditional C5 activation pathways such as the coagulation cascade might also prove beneficial for treating inflammatory arthritis in certain settings.

## Conclusions

The key finding of this study is that autoantibody-mediated arthritis in K/BxN mice can occur via a C5 activation pathway that requires neither C3 nor activating FcγRs, the two main effector mechanisms of IgG molecules. Genetic deficiency of C3, C5, and/or FcRγ did not affect T-cell activation or autoantibody production, indicating that the pro-arthritogenic activity of C5 is mediated by its conventional effector mechanisms (likely C5a production). Our data further suggest that thrombin or related proteases of the coagulation cascade mediate C5 activation in the absence of C3 and FcRγ. Understanding how novel pathways of complement activation contribute to autoantibody-mediated arthritis and other inflammatory disorders is expected to lead to new therapeutic approaches.

## Abbreviations

DMSO: dimethyl sulfoxide; FcγR: Fc receptor for immunoglobulin G; FcRγ: the cytoplasmic signaling chain shared by activating Fc receptors for immunoglobulin G; GPI: glucose-6-phosphate isomerase; IFNγ: interferon-gamma; IgG: immunoglobulin G; IL-17: interleukin-17; LPS: lipopolysaccharide; MAC: membrane attack complex; MHC: major histocompatibility complex; NOD: non-obese diabetic; TCR: T-cell receptor; Th17: T helper 17.

## Competing interests

The authors declare that they have no competing interests.

## Authors' contributions

JLA designed and performed experiments, interpreted data, and assisted in writing the manuscript. SSH performed experiments and assisted in writing the manuscript. BAB designed and oversaw experiments, interpreted data, and wrote the manuscript. All authors read and approved the final manuscript.

## Supplementary Material

Additional file 1 **IgG and C3 deposition in ankles of K/BxN mice lacking C3 or C5 and lacking FcR^©^**. Deposition of IgG (red, left panels) and C3 (green, right panels) was determined by immunofluorescent microscopy in K/BxN mice expressing or not expressing C3, FcR^©^, and C5, as indicated in the left column. The key findings are that IgG is deposited in each of the joints. As expected, C3 is absent in the C3-deficient animal (A, lower right panel). Despite having decreased arthritis severity, C3 is still detectable in the joints of the C5/FcR^©^-deficient mouse (B, lower right panel). Slides were counterstained with DAPI (blue) to detect nuclei. Original objective: 40x. Click here for file

Additional file 2 **Hepatic expression of prothrombin/thrombin is upregulated in C3-deficient mice**. Liver sections from C3-sufficient (left panels) and C3-deficient (right panels) mice were stained with monoclonal antibodies specific for prothrombin/thrombin (top panels) or isotype control antibodies (bottom panels). Brown staining represents bound antibody. The slides were counterstained with hematoxylin (blue). Original objective: 40x. Click here for file
