# Supplementary figures and images for: Autoantibody-mediated arthritis in the absence of C3 and activating Fcγ receptors: C5 is activated by the coagulation cascade
Source: Arthritis Res Ther. 2012 Dec 13;14(6):R269. doi: 10.1186/ar4117 (PMC3674630; doi:10.1186/ar4117)

**Additional File 1**  
**Auger et al.**

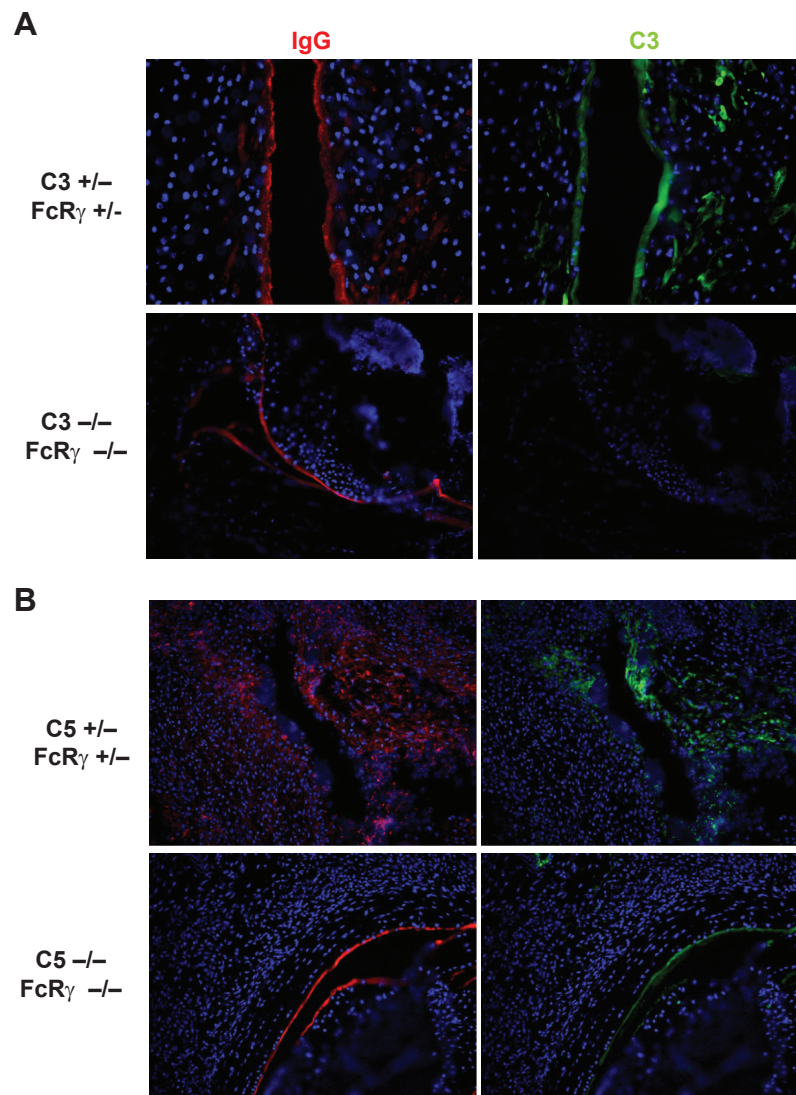

Supplement: Additional file 1 — IgG and C3 deposition in ankles of K/BxN mice lacking C3 or C5 and lacking FcR©. Deposition of IgG (red, left panels) and C3 (green, right panels) was determined by immunofluorescent microscopy in K/BxN mice expressing or not expressing C3, FcR©, and C5, as indicated in the left column. The key findings are that IgG is deposited in each of the joints. As expected, C3 is absent in the C3-deficient animal (A, lower right panel). Despite having decreased arthritis severity, C3 is still detectable in the joints of the C5/FcR©-deficient mouse (B, lower right panel). Slides were counterstained with DAPI (blue) to detect nuclei. Original objective: 40x. [file ar4117-S1.PDF]

**Additional File 2**  
**Auger et al.**

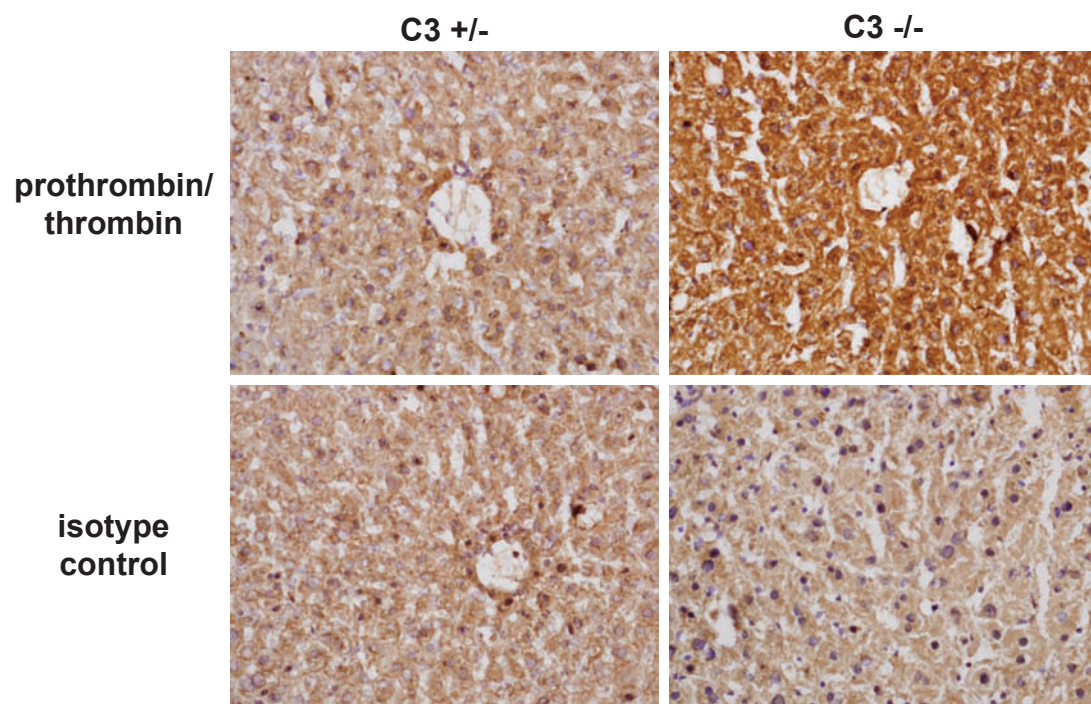

Supplement: Additional file 2 — Hepatic expression of prothrombin/thrombin is upregulated in C3-deficient mice. Liver sections from C3-sufficient (left panels) and C3-deficient (right panels) mice were stained with monoclonal antibodies specific for prothrombin/thrombin (top panels) or isotype control antibodies (bottom panels). Brown staining represents bound antibody. The slides were counterstained with hematoxylin (blue). Original objective: 40x. [file ar4117-S2.PDF]
